# Echocardiographic characterization of combined postcapillary and precapillary pulmonary hypertension in systemic sclerosis with preserved ejection fraction

**DOI:** 10.1186/s44156-026-00116-4

**Published:** 2026-06-08

**Authors:** Ahmad Daoud, Garrett Goldin, Lea Goren, Arindam Bagga, Hoda Mombeini, Ryan Osgueritchian, Vivek P. Jani, Steven Hsu, Fredrick M. Wigley, Matthew R. Lammi, Paul M. Hassoun, Ami A. Shah, Stephen C. Mathai, Monica Mukherjee

**Affiliations:** 1 Division of Cardiology, Johns Hopkins University, 600 N. Wolfe Street Carnegie 536 |, Baltimore, MD 21287 USA; 2 Johns Hopkins University School of Medicine, Baltimore, MD USA; 3 Johns Hopkins University Division of Rheumatology, Baltimore, MD USA; 4 Division of Pulmonary and Critical Care Medicine, Johns Hopkins University, Baltimore, MD USA

**Keywords:** Pulmonary hypertension, Systemic sclerosis, Heart failure with preserved ejection fraction, Echocardiography

## Abstract

**Background:**

Systemic sclerosis (SSc) is a multisystem autoimmune disease frequently complicated by pre- and post-capillary pulmonary hypertension (PH). Within SSc, progressive diastolic dysfunction and heart failure with preserved ejection fraction (HFpEF) are key contributors, often presenting as isolated postcapillary PH (Ipc-PH) or combined pre- and postcapillary PH (Cpc-PH). The ability to differentiate these hemodynamic phenotypes is critical for risk stratification, yet echocardiographic markers specific to each subtype in SSc-HFpEF are poorly defined.

**Methods:**

We investigated 147 adults with SSc-HFpEF with echocardiograms and right heart catheterization (RHC) assessments performed within one year. Patients were classified as Ipc-PH (*n* = 46) or Cpc-PH (*n* = 101) based on guideline-defined hemodynamic criteria. Echocardiographic parameters, including conventional measures, strain indices, and coupling metrics were analyzed. A random forest (RF) classifier was used to identify top echocardiographic predictors of Cpc-PH, and further assessed using multivariable logistic regression. Separate RF models and Cox regression analyses were used to determine echocardiographic predictors of mortality within each group. Survival was assessed using Kaplan-Meier analysis.

**Results:**

Patients with Cpc-PH exhibited significantly greater right heart remodeling, higher pulmonary pressures, and impaired right ventricle (RV)-pulmonary artery (PA) coupling. The top echocardiographic predictors of Cpc-PH included reduced RV free wall strain (RVFWS), decreased RVFWS/PA systolic pressure (PASP) and fractional area change (FAC)/PASP ratios, elevated PASP, lower septal e′ velocity, and higher systolic LV eccentricity index (LV EI). In adjusted Cox models, elevated LVEI (HR 1.39), increased RV internal diastolic diameter (HR 2.15), and reduced left and right atrial strain (HR 0.91 and 0.94, respectively) were independently associated with mortality in Cpc-PH. In Ipc-PH, mortality was linked to reduced FAC/PASP, lower left ventricular global longitudinal strain (LVGLS), increased LV mass index, elevated PASP, and lower RVFWS.

**Conclusion:**

In the present study, we demonstrate key echocardiographic differences between Ipc-PH and Cpc-PH within the SSc-HFpEF population, emphasizing the central role of right heart remodeling, RV-PA coupling, atrial and septal mechanics in phenotypic differentiation and prognostication. Strain-based parameters and RV-PA coupling indices offer incremental value for risk stratification and may guide more tailored therapeutic strategies in this heterogeneous population.

## Background

Systemic sclerosis (SSc) is a complex autoimmune disease characterized by immune dysregulation, vasculopathy and progressive fibrosis of the skin and internal organs [1, 2]. Pulmonary hypertension (PH) across all World Symposium on Pulmonary Hypertension (WSPH) classifications [3], is highly prevalent in SSc, and can develop through several interrelated mechanisms, including pulmonary arterial vasculopathy, interstitial lung disease (ILD)-related hypoxia, and left heart disease (LHD), frequently resulting in mixed hemodynamic profiles [2, 4, 5]. Recent advances in screening, risk stratification, and early initiation of directed therapies [6] have led to marked improvement in mortality and clinical outcomes in SSc-associated pulmonary arterial hypertension (PAH), however, these benefits have not extended to SSc-PH related to LHD [7]. 

LHD is common in SSc, and likely related to diastolic dysfunction, which has an estimated prevalence of 19% [8], and thought to be as an early cardiac manifestation of SSc heart disease, occurring prior to the onset of clinical heart failure [9]. In addition to established cardiovascular comorbidities, such as increasing age, essential hypertension, obesity, diabetes, and coronary artery disease, SSc patients are also affected by disease-specific risk factors, such as SSc disease duration, lower diffusion capacity of carbon monoxide (DL_CO_), anti-Scl-70 positivity, and history of SSc-associated renal crisis and severe gastrointestinal disease [8]. Disease-specific mechanisms, both independently and in conjunction with traditional risk factors, contribute to the increased prevalence of diastolic dysfunction and a higher incidence of heart failure with preserved ejection fraction (HFpEF), both of which are associated with a four-fold increase in mortality [8–10]. 

PH-LHD is a major contributor to morbidity and mortality in SSc, particularly among patients with HFpEF, where it accounts for 20–45% of SSc-associated PH cases [11]. In SSc-HFpEF, patients may exhibit either isolated post-capillary PH (Ipc-PH) or combined pre- and post-capillary PH (Cpc-PH). In some patients with SSc-PAH receiving PAH-directed therapies, overlapping clinical features of HFpEF may emerge over time, potentially reflecting mixed pathophysiologic contributions, as seen in the Cpc-PH phenotype. Distinguishing Ipc-PH from Cpc-PH in SSc patients with PH-LHD remains particularly challenging, given the substantial clinical and hemodynamic overlap between these entities [12]. 

Echocardiography is a key diagnostic tool in the screening and early detection of emerging pulmonary vascular disease (PVD) in patients with SSc [13, 14]. However, the role of echocardiography in differentiating relevant hemodynamic and structural differences between Ipc-PH and Cpc-PH phenotypes in SSc is unclear. In the present study, we compared the echocardiographic characteristics of Cpc-PH and Ipc-PH within a comprehensively profiled SSc-HFpEF population. We hypothesized that distinct echocardiographic features differentiate these two phenotypes, thereby refining clinical phenotyping and improving risk stratification. Enhanced noninvasive phenotyping of Cpc-PH may also inform the need for confirmatory right heart catheterization (RHC), facilitate earlier referral to specialty PH programs, and guide management decisions.

## Methods

### Study population

Adult patients with SSc enrolled in the Johns Hopkins Scleroderma Center and Johns Hopkins Pulmonary Hypertension Program who fulfilled the American College of Rheumatology and European League Against Rheumatism scleroderma classification criteria [15, 16] and had invasive hemodynamic confirmation of Ipc-PH and Cpc-PH status within one year of transthoracic echocardiography (TTE) were included in this cohort study. The interval between TTE and RHC was reported as the absolute number of days between study dates. The onset of SSc was defined as the time of the first non-Raynaud manifestation attributable to the disease. HFpEF was defined as pulmonary capillary wedge pressure (PCWP) > 15 mmHg, measured either at rest or through standardized exercise provocation using supine bicycle ergometry during clinical RHC hemodynamic assessment, along with a left ventricular ejection fraction (LVEF) > 50% on TTE. While not all patients underwent exercise testing, those who exceeded the PCWP threshold during exercise were included in the final cohort. For patients who underwent exercise RHC, we recorded peak mean pulmonary artery pressure (mPAP) and PCWP, and calculated the mPAP/cardiac output (CO) slope from rest to peak exercise [17]. Pulmonary function tests (PFTs), six-minute walk distance (6MWD), laboratory values, and PH-specific therapies actively prescribed at the time of echocardiography were collected when available, using results from studies performed closest to the date of echocardiography. All patients met updated WSPH diagnostic criteria for PH-LHD [3], defined by mPAP > 20 mmHg and PCWP > 15 mmHg, and further classified into Ipc-PH and Cpc-PH subgroups. Ipc-PH was defined by a PCWP > 15 mmHg at rest or during provocation and a pulmonary vascular resistance (PVR) ≤ 2 Wood Units (WU). Cpc-PH was defined by a PCWP > 15 mm [3]. 

Survival was assessed from the date of echocardiography until the date of death or censoring, with patients censored at their last known clinical follow-up as of March 1, 2025. Outcomes were determined through review of the electronic health record. The study was approved by the Johns Hopkins University Institutional Review Board. Written informed consent was obtained from all participants upon entry into the registry.

### Echocardiographic methods

Technically adequate echocardiograms performed at the Johns Hopkins Medical Institutions, Baltimore, Maryland within one year of RHC were included. Linear and volumetric measurements of the cardiac chambers were performed following American Society of Echocardiography (ASE) guidelines using standardized analytic software (FujiFilm ProSolve 4.0, Indianapolis, Indiana) at a frame rate of 70–90 frames per second [13, 18]. Volumetric measures were adjusted to body surface area (BSA). Conventional RV functional measures included RV FAC (%), tissue Doppler imaging (TDI) peak S’ velocity (cm/s), RV outflow tract (RVOT) velocity-time integral (VTI), and M-mode derived TAPSE (mm). The left ventricular eccentricity index (LVEI) was measured at end-systole in the parasternal short-axis view at the level of the papillary muscles, calculated as the ratio of the LV anteroposterior diameter to the septolateral diameter. The modified Bernoulli equation was used to calculate RV systolic pressure (RVSP, mmHg) from the peak tricuspid regurgitant (TR) velocity. In the absence of pulmonic stenosis or RVOT obstruction, pulmonary artery systolic pressure (PASP, mmHg) was calculated by RVSP added to the right atrial pressure (RAP) as estimated by inferior vena cava diameter and collapsibility with respiration.

Speckle-tracking echocardiography (STE) was performed using vendor-independent software (Epsilon EchoInsight 3.2, Ann Arbor, Michigan) to evaluate chamber-level mechanics and myocardial deformation [19]. Strain analysis included all four cardiac chambers: left atrial (LA) reservoir strain, left ventricular global longitudinal strain (LVGLS), right atrial (RA) reservoir strain, and 3-segment RV free wall strain (RVFWS). Ventricular strain values (LVGLS and RVFWS) were expressed as negative percentages, reflecting myocardial fiber shortening during systolic contraction. In contrast, atrial reservoir strain values were expressed as positive percentages, representing chamber expansion during ventricular systole. LVGLS was calculated from three standard apical views, encompassing 18 myocardial segments. RVFWS was calculated as the average peak strain from the basal, mid, and apical segments of the RV free wall. Strain values from all chambers were compared to established reference values [19, 20]. 

Echo-derived RV-PA coupling ratios included TAPSE/PASP (mm/mm Hg), FAC/PASP (%/mm Hg), and 3-segment RVFWS/PASP (%/mm Hg). In addition, atrioventricular strain coupling ratios were also assessed to evaluate the relationship between atrial and ventricular function. Specifically, RA/RV and LA/LV strain ratios were calculated by dividing atrial reservoir strain by the corresponding ventricular longitudinal strain (RA strain/RVFWS and LA strain/LVGLS), providing a measure of atrial-ventricular mechanical interaction and potential uncoupling. Echocardiograms were read by two independent readers blinded to hemodynamics and clinical parameters and the intraclass correlation (ICC) was calculated to assess the agreement between echocardiographic measurements obtained in this study.

### Statistical analysis

Clinical and echocardiographic characteristics of participants were summarized as mean ± standard deviation (SD) for continuous variables. Normality of data distribution was assessed using the Shapiro-Wilk test. Normally distributed variables were compared using independent sample t-tests, while non-normally distributed variables were analyzed using the Mann-Whitney U test. Categorical variables were compared using chi-square tests. Descriptive analyses were performed on complete case data without imputation. To identify key echocardiographic features distinguishing Cpc-PH from Ipc-PH, machine learning-based variable selection was conducted using a random forest (RF) classifier. Missing data were handled using the MissForest imputation method, which is a nonparametric, iterative random forest-based technique capable of handling both continuous and categorical variables [21]. The model was trained to classify patients as either Cpc-PH or Ipc-PH using all echocardiographic parameters, and the top 10 predictors were selected based on variable importance rankings. The top 10 echocardiographic predictors identified by the RF model were analyzed using logistic regression. Both unadjusted and multivariable-adjusted models were constructed. The adjusted model included covariates for age (defined as age at the time of echocardiography), sex, and SSc disease duration, which was defined as the time from the first non-Raynaud’s symptom attributable to SSc to the date of echocardiography. To evaluate mortality risk factors within each hemodynamic phenotype, two separate RF models were trained to identify the top five echocardiographic predictors of death in the Ipc-PH and Cpc-PH subgroups, respectively. These models were run on MissForest-imputed datasets, and predictors were selected based on variable importance rankings. For each group, the top five mortality predictors identified by the respective RF models were subsequently analyzed using Cox proportional hazards regression. Both unadjusted and multivariable-adjusted models were constructed. Adjusted models included covariates for age, sex, and SSc disease duration.

Due to the early crossover of Kaplan-Meier survival curves, restricted mean survival time (RMST) analysis was applied to compare survival between groups. RMST was calculated over a 10-year (120-month) horizon as the area under the Kaplan-Meier curve. Standard errors for each RMST estimate were computed to facilitate between-group comparisons using a two-sample t-test. Kaplan-Meier survival curves were generated in Python (v3.10) using the lifelines package, with an accompanying table displaying the number of patients at risk at defined timepoints. All statistical analyses were performed using Stata (Version 18.5, College Station, TX) and Python Software Foundation (Python Language Reference, version 3.10. 2021. https://www.python.org). Statistical significance was defined by a 2-sided p-value < 0.05.

## Results

### Clinical characteristics of the study cohort

Among 147 patients with SSc-HFpEF, 46 were classified as Ipc-PH and 101 as Cpc-PH by invasive hemodynamics, as shown in Table 1. The median interval between RHC and TTE was 97 days (IQR 26–270). Patients with Cpc-PH tended to be older than those with Ipc-PH (61.8 ± 10.8 vs. 58.3 ± 11.9 years, *p* = 0.079) and were more frequently female (83.2% vs. 69.6%, *p* = 0.061). Race and body mass index (BMI) distributions were similar between groups. Most patients were White, although the Ipc-PH group had a slightly higher proportion of Black patients (28.3% vs. 16.8%). SSc disease duration and mortality did not significantly differ between groups. Limited cutaneous SSc was the predominant diagnosis; however, mixed connective tissue disease with SSc-predominant features was more frequently observed in the Cpc-PH group, while SSc sine scleroderma was more frequent in the Ipc-PH group (*p* = 0.038). The prevalence of any telangiectasia was significantly higher among patients with Cpc-PH compared to those with Ipc-PH (91.1% vs. 78.3%, *p* = 0.032). Among autoantibody profiles, ACA positivity was significantly higher in the Cpc-PH group (36.6% vs. 17.4%, *p* = 0.019), while frequencies of ANA, Scl-70, RNA polymerase III, and RNP antibodies were similar between groups. Cardiometabolic comorbidities were prevalent in both groups, with no statistically significant differences observed. Although the prevalence of ILD, defined by the presence of fibrotic changes evident on high-resolution computed tomography (HRCT), was similar between groups, patients with Cpc-PH had significantly lower forced vital capacity (FVC) (72.0 ± 19.3% vs. 78.5 ± 16.3% predicted, *p* = 0.049) and diffusing capacity for carbon monoxide (DL_CO_) (54.8 ± 21.8% vs. 65.4 ± 24.1% predicted, *p* = 0.014) compared to those with Ipc-PH. There were no significant differences in other PFT parameters.

**Table 1:** Baseline characteristics, systemic sclerosis features, comorbidities, pulmonary function and hemodynamics

Demographics	Ipc-PH (*n* = 46)	Cpc-PH (*n* = 101)	*p*-value
Age, years	58.3 ± 11.9	61.8 ± 10.8	0.079
Sex, n (%)			
Female Male	32 (69.6%) 14 (30.4%)	84 (83.2%) 17 (16.8%)	0.061
Death, n (%)	21 (45.7%)	55 (54.5%)	0.322
SSc Disease Duration, yrs ± SD	7.7 ± 8.7	9.4 ± 8.6	0.264
Race, n (%)			
White Black Other	28 (60.9%) 13 (28.3%) 5 (10.9%)	78 (77.2%) 17 (16.8%) 6 (5.9%)	0.536
BMI (kg/m²), mean ± SD	28.0 ± 7.6	28.4 ± 6.6	0.732
Systemic Sclerosis Characteristics			
SSc Diagnosis, n (%)			
Limited cutaneous Diffuse cutaneous SSc Sine scleroderma MCTD	26 (56.5%) 5 (10.9%) 12 (26.1%) 3 (6.5%)	67 (66.3%) 8 (7.9%) 9 (8.9%) 14 (13.9%)	**0.038**
Telangiectasia	36 (78.3%)	92 (91.1%)	**0.032**
SSc related antibodies, n (%)			
ANA Positive ACA Positive Scl-70 Positive RNA Polymerase III Positive RNP Antibody Positive	32 (69.6%) 8 (17.4%) 12 (26.1%) 9 (19.6%) 2 (4.4%)	84 (83.2%) 37 (36.6%) 19 (18.8%) 14 (14.1%) 10 (9.9%)	0.061 **0.019** 0.316 0.405 0.254
Cardiopulmonary and Systemic Comorbidities, n (%)			
Diabetes Mellitus	12 (26.1%)	18 (17.8%)	0.249
Systemic Hypertension	40 (87.0%)	77 (76.2%)	0.135
Coronary Artery Disease	17 (37.0%)	25 (24.8%)	0.136
Atrial Fibrillation	16 (34.8%)	31 (30.7%)	0.622
ILD	30 (65.2%)	59 (59.0%)	0.474
Pulmonary Hypertension-Specific Therapies, n (%)			
PDE5 Inhibitor	20 (43.5%)	52 (51.5%)	0.368
Endothelin Receptor Antagonist	5 (10.9%)	36 (35.6%)	**0.002**
Prostacyclin Analogs	4 (8.7%)	22 (22.0%)	**0.048**
Pulmonary Function Tests, mean ± SD (n)			
FVC, % predicted	78.5 ± 16.3 (46)	72.0 ± 19.3 (99)	**0.049**
FEV1, % predicted	77.2 ± 15.5 (43)	69.6 ± 18.6 (97)	**0.022**
FEV1/FVC ratio, %	79.4 ± 11.2 (45)	77.7 ± 8.6 (94)	0.316
TLC, % predicted	80.0 ± 19.8 (42)	75.5 ± 18.1 (78)	0.208
DLCO, % predicted	65.4 ± 24.1 (41)	54.8 ± 21.8 (88)	**0.014**
RV, % predicted	81.3 ± 35.9 (38)	79.3 ± 27.9 (74)	0.737
6-Minute Walk Test, mean ± SD (n)			
Distance, meters	350.1 ± 85.4 (21)	304.9 ± 129.8 (75)	0.137
% Predicted	72.2 ± 17.5(18)	62.7 ± 24.5 (74)	0.126
Hemodynamic Parameters, mean ± SD (n)			
Heart Rate, bpm	75.9 ± 13.6 (39)	78.1 ± 16.9 (91)	0.497
SBP, mmHg	133.7 ± 19.5 (39)	137.4 ± 26.6 (92)	0.427
DBP, mmHg	74.0 ± 9.4 (39)	75.3 ± 12.8 (92)	0.578
RAP, mmHg	9.3 ± 6.4 (45)	9.9 ± 4.5 (98)	0.536
mPAP rest, mmHg mPAP exercise, mmHg	25.9 ± 6.4 (46) 34.6 ± 4.9 (8)	38.1 ± 12.7 (101) 38.6 ± 6.8 (16)	**0.001** 0.162
PCWP rest, mmHg PCWP exercise, mmHg	17.8 ± 4.9 (46) 22.4 ± 5.1 (8)	16.9 ± 4.5 (101) 19.0 ± 3.8 (16)	0.282 0.081
Resting RAP/PCWP	0.52 ± 0.29 (45)	0.59 ± 0.23 (98)	**0.047**
PVR, WU	1.4 ± 0.5 (46)	4.7 ± 3.3 (101)	
CO, L/min1	5.9 ± 2.2 (44)	4.8 ± 1.2 (96)	**0.001**
CI, L/min/m²	3.2 ± 1.1 (44)	2.6 ± 0.6 (97)	**0.001**
mPAP/CO slope, mmHg/L/min	4.9 ± 2.8 (8)	4.4 ± 2.8 (12)	0.701

This table compares demographic data, systemic sclerosis (SSc) characteristics, comorbid conditions, medication use, pulmonary function tests (PFTs), 6-minute walk distance (6MWD), and right heart catheterization (RHC)-derived hemodynamics between patients with isolated post-capillary pulmonary hypertension (Ipc-PH) and those with combined pre- and post-capillary PH (Cpc-PH). Continuous variables are presented as mean ± standard deviation (SD), with the number of individuals (n) in parentheses where applicable. Categorical variables are reported as counts and percentages. Group comparisons were made using t-tests for continuous variables and chi-square tests for categorical variables. Bolded values indicate statistically significant p-values (*p* < 0.05)

Abbreviations: ACA = anticentromere antibody; AF = atrial fibrillation; ANA = antinuclear antibody; BMI = body mass index; CAD = coronary artery disease; CI = cardiac index; CO = cardiac output; Cpc-PH = combined pre- and post-capillary pulmonary hypertension; DBP = diastolic blood pressure; DLCO = diffusing capacity for carbon monoxide; FVC = forced vital capacity; FEV1 = forced expiratory volume in 1 s; HR = heart rate; ILD = interstitial lung disease; Ipc-PH = isolated post-capillary pulmonary hypertension; MCTD = mixed connective tissue disease; mPAP = mean pulmonary artery pressure; PCWP = pulmonary capillary wedge pressure; PDE5i = phosphodiesterase-5 inhibitor; PFT = pulmonary function test; PVR = pulmonary vascular resistance; RAP = right atrial pressure; RNP = ribonucleoprotein antibody; RV= Residual Volume; SBP = systolic blood pressure; Scl-70 = anti-topoisomerase I antibody; SSc = systemic sclerosis; TLC = total lung capacity; 6MWD = 6-minute walk distance; WU = Wood units

RHC demonstrated key hemodynamic differences between groups. The mPAP was significantly higher in Cpc-PH (38.1 ± 12.7 vs. 25.9 ± 6.4 mmHg, *p* = 0.001), and despite preserved left ventricular (LV) systolic function, patients with Cpc-PH exhibited significantly lower resting cardiac output (4.8 ± 1.2 vs. 5.9 ± 2.2 L/min, *p* = 0.001) and cardiac index (2.6 ± 0.6 vs. 3.2 ± 1.1 L/min/m^2^, *p* = 0.001). Resting RAP/PCWP ratio, assessing the balance and relationship between right atrial and left-sided filling pressures, was also significantly higher in Cpc-PH compared to Ipc-PH (0.59 ± 0.23 vs. 0.52 ± 0.29, *p* = 0.047). Other parameters, including RAP, heart rate, systemic blood pressure, and PCWP at rest, did not differ significantly between groups.

### Echocardiographic characteristics

Echocardiographic characteristics stratified by hemodynamic group are presented in Table 2. Compared to the Cpc-PH group, patients with Ipc-PH had significantly larger LV end-diastolic and end-systolic chamber diameters (*p* = 0.016 and *p* = 0.015, respectively). Furthermore, patients with Ipc-PH had a significantly higher LV mass index (95.8 ± 51.3 vs. 84.8 ± 21.8 g/m^2^, *p* = 0.036). In contrast, Cpc-PH patients demonstrated significantly higher systolic LVEI (1.09 ± 0.22 vs. 0.95 ± 0.14, *p* = 0.004), reflecting greater interventricular septal flattening and RV overload. Cpc-PH patients also exhibited a greater LA area (19.1 ± 4.7 vs. 16.6 ± 3.8 cm^2^, *p* = 0.017). Diastolic function was more impaired in Cpc-PH, as reflected by significantly lower septal e′ velocities (6.9 ± 2.0 vs. 8.4 ± 2.5 cm/s, *p* = 0.002) and reduced lateral e′ velocities (8.7 ± 2.8 vs. 10.3 ± 2.9 cm/s, *p* = 0.049).

**Table 2:** Key echocardiographic parameters

Parameter	Ipc-PH (Mean ± SD, *N*)	Cpc-PH (Mean ± SD, *N*)	*p*-value
LV EF, %	62.2 ± 6.2 (45)	61.4 ± 5.0 (101)	0.410
LVEDD, cm	4.6 ± 0.8 (45)	4.3 ± 0.6 (98)	**0.016**
LVESD, cm	3.0 ± 0.6 (42)	2.8 ± 0.6 (94)	**0.015**
LV Mass Index, (g/m²)	95.8 ± 51.3 (44)	84.8 ± 21.8 (98)	**0.036**
Mitral E/A ratio	1.2 ± 0.5 (38)	1.1 ± 0.5 (85)	0.387
E’ Septal, cm/s	8.4 ± 2.5 (34)	6.9 ± 2.0 (76)	**0.002**
E’ Lateral, cm/s	10.3 ± 2.9 (19)	8.7 ± 2.8 (34)	**0.049**
E/E’ Septal Ratio	13.2 ± 13.0 (33)	14.1 ± 7.0 (77)	0.625
E/E’ Lateral Ratio	10.8 ± 10.7 (19)	11.4 ± 9.2 (34)	0.830
LA Area, cm²	16.6 ± 3.8 (27)	19.1 ± 4.7 (64)	**0.017**
LAVi, ml/m²	28.8 ± 13.4 (41)	29.8 ± 13.1 (93)	0.674
LA Diameter, cm	3.8 ± 0.9 (41)	3.8 ± 0.7 (98)	0.926
End-systolic LVEI	0.95 ± 0.14 (24)	1.09 ± 0.22 (73)	**0.004**
RVIDD, cm	3.0 ± 0.7 (32)	3.4 ± 0.6 (88)	**0.002**
Mid RVOT Diameter, cm	3.4 ± 0.7 (24)	3.5 ± 0.5 (74)	0.214
RVOT VTI, cm	14.1 ± 3.6 (13)	13.4 ± 2.6 (35)	0.486
RA Area, cm²	16.0 ± 6.2 (33)	18.8 ± 6.7 (94)	**0.039**
RAVI, ml/m²	23.8 ± 16.1 (32)	30.6 ± 16.8 (91)	**0.048**
RV Basal Diameter, cm	3.9 ± 0.6 (31)	4.3 ± 0.8 (89)	**0.006**
RV Mid Diameter, cm	2.9 ± 0.7 (31)	3.5 ± 0.8 (83)	**0.001**
RV Apical Diameter, cm	7.9 ± 0.9 (28)	8.1 ± 1.1 (79)	0.405
RVEDA, cm²	19.5 ± 4.4 (27)	22.1 ± 6.9 (77)	0.061
RVESA, cm²	10.8 ± 3.5 (27)	13.5 ± 6.0 (78)	**0.029**
FAC, %	45.2 ± 11.3 (27)	40.1 ± 11.0 (77)	0.091
TAPSE, mm	20.76 ± 5.27 (24)	19.36 ± 4.40 (60)	0.217
TDI S’ Velocity, cm/s	12.4 ± 2.4 (11)	11.8 ± 3.1 (27)	0.712
TR Peak Velocity, m/sec	2.8 ± 0.5 (36)	3.3 ± 0.7 (90)	**0.001**
PASP, mmHg	37.5 ± 12.1 (39)	54.4 ± 23.6 (91)	**0.001**
FAC/PASP, %/mmHg	1.33 ± 0.61 (22)	1.01 ± 0.66 (70)	**0.044**
TAPSE/PASP, mm/%	0.62 ± 0.32 (19)	0.46 ± 0.25 (54)	**0.030**
Left Atrial Reservoir Strain, %	28.0 ± 7.2 (27)	25.3 ± 12.3 (71)	0.287
LVGLS, %	-20.0 ± 2.1 (24)	-19.5 ± 5.5 (57)	0.674
Left Atrial Reservoir Strain / LVGLS, %	1.39 ± 0.35 (20)	1.27 ± 0.58 (53)	0.370
RA Reservoir Strain, %	32.9 ± 8.6 (24)	31.1 ± 9.0 (56)	0.411
Global RVFWS, %	-23.3 ± 4.5 (22)	-20.7 ± 4.7 (60)	**0.032**
RA Reservoir Strain / RVFWS, %	1.54 ± 0.53 (20)	1.52 ± 0.38 (48)	0.823
RVFWS/PASP, %/mmHg	0.77 ± 0.33 (17)	0.55 ± 0.32 (54)	**0.013**

Bolded values indicate statistically significant p-values (*p* < 0.05)

Abbreviations: Cpc-PH = combined pre- and post-capillary pulmonary hypertension; FAC = fractional area change (right ventricle); Ipc-PH = isolated post-capillary pulmonary hypertension; LA = Left Atrium; LAVi = left atrial volume indexed to body surface area; LVEDD = left ventricular end-diastolic diameter; LVEF = left ventricular ejection fraction; LVEI = left ventricular eccentricity index (end-systole); LVESD = left ventricular end-systolic diameter; PASP = Pulmonary Artery Systolic Pressure (estimated via echocardiography); RA Area = Right atrial area; RAVI = right atrial volume index; RVEDA = right ventricular end-diastolic area; RVESA = right ventricular end-systolic area; RVFWS = right ventricular free-wall strain (average of three segments); RVIDD = right ventricular internal diameter in diastole; RVOT VTI = right ventricular outflow tract velocity–time integral; TAPSE = tricuspid annular plane systolic excursion; TDI S′ Velocity = tissue Doppler imaging peak systolic velocity; TR = tricuspid regurgitation

**Fig. 1:**
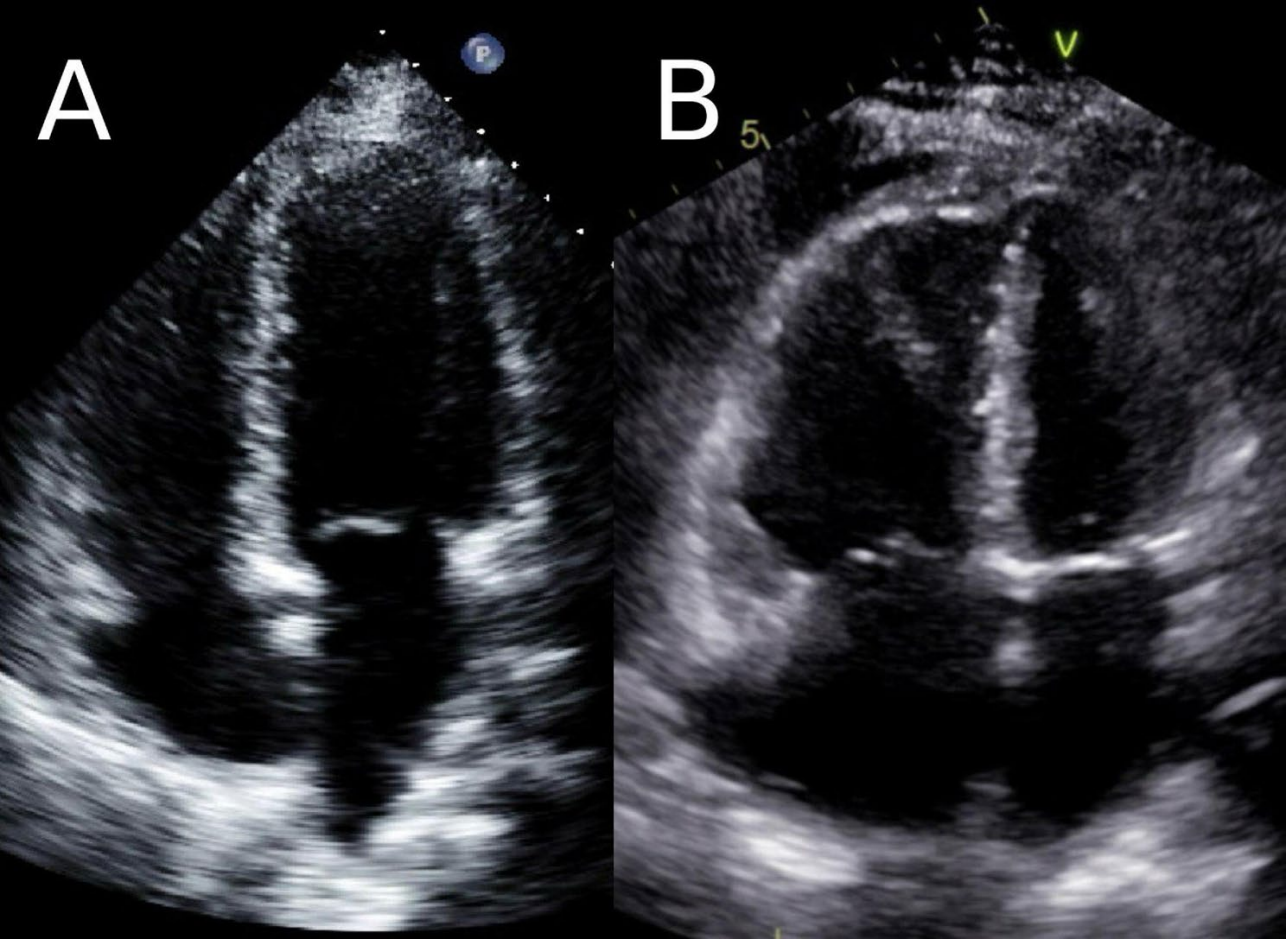
Representative echocardiographic images from patients with systemic sclerosis-associated heart failure with preserved ejection fraction (SSc-HFpEF). (**A**) Isolated postcapillary pulmonary hypertension (Ipc-PH) phenotype. (**B**) Combined pre- and post-capillary pulmonary hypertension (Cpc-PH) phenotype, showing larger right atrial and right ventricular chamber dimensions

Right heart size and function also differed between groups. Patients with Cpc-PH had significantly larger RV linear dimensions, including basal diameter (4.3 ± 0.8 vs. 3.9 ± 0.6 cm, *p* = 0.006) and midventricular diameter (3.5 ± 0.8 vs. 2.9 ± 0.7 cm, *p* = 0.001). The RA chamber size was also larger in Cpc-PH patients, as reflected by a higher RA volume index (RAVI, 30.6 ± 16.8 vs. 23.8 ± 16.1 ml/m^2^, *p* = 0.048) and greater RA area (18.8 ± 6.7 vs. 16.0 ± 6.2 cm^2^, *p* = 0.039), as qualitatively illustrated in Fig. 1. Noninvasive hemodynamic estimates of pulmonary pressures were also significantly higher in Cpc-PH, with increased peak TR velocity (3.3 ± 0.7 vs. 2.8 ± 0.5 m/s, *p* = 0.001) and PASP (54.4 ± 23.6 vs. 37.5 ± 12.1 mmHg, *p* = 0.001). While TAPSE and FAC were not significantly different between groups, RVFWS was significantly lower (-20.7 ± 4.7% vs. -23.3 ± 4.5%, *p* = 0.032) in the Cpc-PH group, as were RV-PA coupling ratios including lower RVFWS/PASP ratios (0.55 ± 0.32 vs. 0.77 ± 0.33, *p* = 0.013) and TAPSE/PASP ratio (0.46 ± 0.25 vs. 0.62 ± 0.32, *p* = 0.030). In the small subset with exercise hemodynamics (*n* = 24), resting echocardiographic indices were similar between Ipc-PH and Cpc-PH. The overall ICC showed close agreement between the readers [0.93 (0.79–0.96)].

### Top echocardiographic predictors of Cpc-PH vs. Ipc-PH

**Fig. 2:**
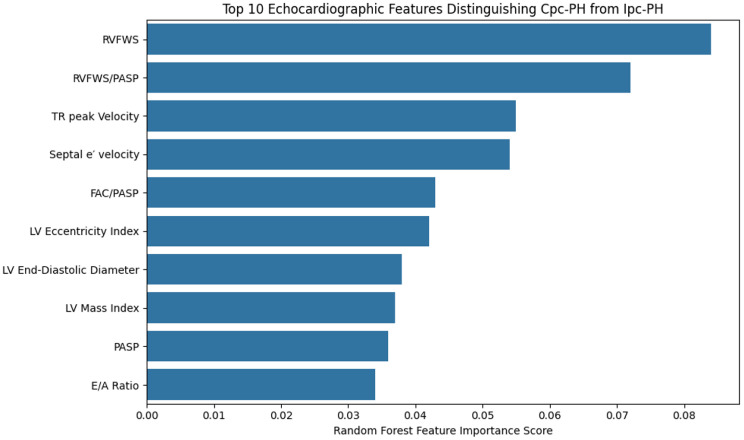
Feature importance rankings derived from a random forest classification model to identify top echocardiographic features distinguishing combined pre- and post-capillary pulmonary hypertension (Cpc-PH) from isolated post-capillary pulmonary hypertension (Ipc-PH) in patients with systemic sclerosis-associated heart failure with preserved ejection fraction (SSc-HFpEF)

To identify top echocardiographic features most strongly associated with Cpc-PH compared to Ipc-PH, a RF classification model was applied to the imputed dataset. The top 10 predictors identified by the model are illustrated in Fig. 2. The top six predictors identified included RVFWS, RVFWS/PASP, TR peak velocity, septal e′ velocity, FAC/PASP, and LVEI. Multivariable logistic regression was then performed separately for each of these predictors as well as additional echocardiographic predictors, adjusting for age, sex, and SSc disease duration. Multivariable-adjusted odds ratios and significance are summarized in Table 3. A lower RVFWS remained the most robust independent predictor (adjusted OR: 1.22, 95% CI: 1.10–1.36, *p* = 0.001), along with lower RVFWS/PASP (adjusted OR: 0.05, 95% CI: 0.01–0.26, *p* = 0.001), reflecting impaired RV-PA coupling among Cpc-PH patients. Higher TR peak velocity, per 0.1 m/s increase, was also strongly associated (adjusted OR: 1.17, 95% CI: 1.08–1.26, *p* = 0.001), as was higher PASP, per 5 mmHg (adjusted OR: 1.33, 95% CI: 1.15–1.54, *p* = 0.001). Lower septal e′ velocity (adjusted OR: 0.75, 95% CI: 0.60–0.91, *p* = 0.004) and higher systolic LVEI, per 0.1-unit increase (adjusted OR: 1.64, 95% CI: 1.25–2.16, *p* = 0.001), were also independently associated with Cpc-PH, further distinguishing this phenotype from Ipc-PH.

**Table 3:** Echocardiographic predictors of Cpc-PH vs. Ipc-PH

Predictor	Adjusted OR (95% CI)	*p*-value
E’ Septal Velocity, m/s*	0.75 (0.60–0.91)	**0.004**
Mitral E/A ratio *	0.78 (0.35–1.71)	0.527
End-systolic LVEI *	1.64 (1.25–2.16)	**0.001**
LV Mass Index *	0.92 (0.89–0.95)	**0.046**
LVEDD, cm*	0.51 (0.29–0.90)	**0.021**
RA Area, cm^2^	1.11 (1.03–1.19)	**0.007**
RAVi, mL/m^2^	1.04 (1.01–1.07)	**0.012**
RV Basal Diameter, cm	3.93 (1.90–5.11)	**0.001**
RV Mid Diameter, cm	3.98 (2.04–7.74)	**0.001**
RV Apical Diameter, cm	1.67 (1.07–2.61)	**0.025**
FAC, %	0.93 (0.89–0.98)	**0.002**
TAPSE, mm	0.89 (0.81–0.98)	**0.016**
TR Peak Velocity, m/s*	1.17 (1.08–1.26)	**0.001**
PASP, mmHg*	1.33 (1.15–1.54)	**0.001**
TAPSE/PASP, mm/mmHg	0.08 (0.02–0.39)	**0.002**
FAC/PASP, %/mmHg	0.28 (0.13–0.60)	**0.001**
LA Reservoir Strain, %	0.95 (0.90–0.96)	**0.045**
RA Reservoir Strain, %	0.93 (0.88–0.98)	**0.007**
RVFWS, %	1.22 (1.10–1.36)	**0.001**
RVFWS/PASP, %/mmHg	0.05 (0.01–0.26)	**0.001**

Odds ratios (OR) and 95% confidence intervals (CI) represent the likelihood of classification as combined pre- and post-capillary pulmonary hypertension (Cpc-PH) over isolated post-capillary PH (Ipc-PH) based on echocardiographic features. All models were adjusted for age at echocardiogram, sex, and systemic sclerosis (SSc) disease duration from first symptom onset. Bolded values indicate statistically significant p-values (*p* < 0.05). Variables marked with an asterisk (*) were among the top 10 most important predictors identified using the random forest classification model

Abbreviations: FAC = fractional area change; LV = left ventricle; LVEI = left ventricular eccentricity index (in systole); LVEDD = left ventricular end-diastolic diameter; PASP = pulmonary artery systolic pressure (estimated via echocardiography); RA Area = right atrial area; RAVi = right atrial volume indexed to body surface area; RVFWS = right ventricular free-wall strain (average of three segments); TAPSE = tricuspid annular plane systolic excursion; TR = tricuspid regurgitation

Beyond these top predictors, additional echocardiographic features also remained significantly associated with Cpc-PH. These included enlarged RV basal (adjusted OR: 3.93, *p* < 0.001) and mid-cavity diameters (adjusted OR: 3.98, *p* < 0.001), larger RA area and volume index (adjusted ORs: 1.11 and 1.04, respectively), and worsened RV systolic function reflected by reduced FAC (adjusted OR: 0.93, *p* = 0.002). Other measures of RV-PA coupling were also lower in Cpc-PH, including TAPSE/PASP (adjusted OR: 0.08, *p* = 0.002) and FAC/PASP (adjusted OR: 0.28, *p* = 0.001). Atrial chamber mechanics also contributed to phenotypic differentiation, with lower RA reservoir strain (adjusted OR: 0.93, *p* = 0.007) and LA reservoir strain (adjusted OR: 0.95, *p* = 0.045) being associated with Cpc-PH.

### Kaplan-meier survival analysis by hemodynamic group

**Fig. 3:**
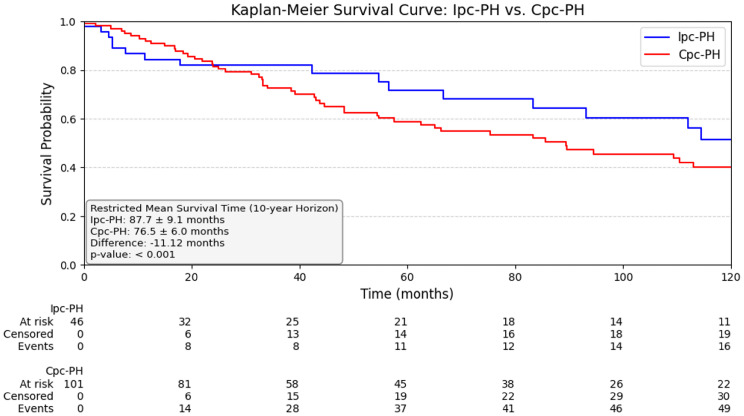
Survival probabilities over 10 years are shown for patients with isolated postcapillary pulmonary hypertension (Ipc-PH) and combined pre-/postcapillary pulmonary hypertension (Cpc-PH) among the systemic sclerosis-associated heart failure with preserved ejection fraction (SSc-HFpEF) population. The figure includes a risk table showing numbers at risk, censored, and events at defined time points. A summary box displays RMST values over a 10-year horizon

Kaplan-Meier survival analysis was performed to compare all-cause mortality between Ipc-PH and Cpc-PH patients within the SSc-HFpEF cohort and to determine whether distinct echocardiographic profiles were associated with mortality. As shown in Fig. 3, survival curves demonstrated early crossover and progressive separation over time, with numerically lower survival in the Cpc-PH group throughout follow-up. The median follow-up time was 4.2 years (IQR: 1.3–6.4) for Ipc-PH and 4.0 years (IQR: 2.1–6.3) for Cpc-PH patients. Due to the violation of the proportional hazards assumption, RMST analysis over a 10-year follow-up was used as the primary comparative method. The mean RMST was 87.7 ± 9.1 months for Ipc-PH and 76.5 ± 6.0 months for Cpc-PH, corresponding to a between-group difference of -11.1 months (*p* < 0.001). These findings reinforce the Kaplan-Meier trends and highlight the statistically significant survival disadvantage among Cpc-PH patients.

### Random forest-based mortality prediction and cox regression

Separate RF models were used to identify the top echocardiographic predictors of mortality in the Ipc-PH and Cpc-PH patient groups. In the Ipc-PH group, the top five predictors of mortality were FAC/PASP ratio, LV mass index, LVGLS, PASP, and RVFWS. Adjusted Cox regression confirmed that lower FAC/PASP was independently associated with increased mortality (adjusted HR: 0.20, 95% CI: 0.05–0.80, *p* = 0.023), as was higher LV mass index (HR: 1.01, 95% CI: 1.01–1.03, *p* = 0.037), lower LVGLS (HR: 1.37, 95% CI: 1.09–1.71, *p* = 0.006), higher PASP (HR: 1.11, 95% CI: 1.05–1.18, *p* = 0.001), and lower RVFWS (HR: 1.28, 95% CI: 1.10–1.48, *p* = 0.001). In the Cpc-PH group, the top predictors included: LVEI, RV internal diastolic diameter (RVIDD), LA reservoir strain, RA reservoir strain, and FAC/PASP ratio. In multivariable Cox models, higher LVEI was associated with increased mortality (adjusted HR: 1.39, 95% CI: 1.07–1.82, *p* = 0.015), along with larger RVIDD (HR: 2.15, 95% CI: 1.26–3.69, *p* = 0.005), lower LA reservoir strain (HR: 0.91, 95% CI: 0.85–0.96, *p* = 0.001), lower RA reservoir strain (HR: 0.94, 95% CI: 0.91–0.98, *p* = 0.003), and reduced FAC/PASP ratio (HR: 0.49, 95% CI: 0.25–0.96, *p* = 0.039).

## Discussion

In the present study, we sought to determine whether distinct echocardiographic features could differentiate a Cpc-PH phenotype from Ipc-PH in a well-characterized cohort of patients with SSc-HFpEF and postcapillary PH-LHD. Our findings demonstrate that patients with Cpc-PH exhibit a more adverse echocardiographic phenotype, characterized by RV maladaptation, elevated pulmonary pressures, and impaired RV-PA coupling. The most discriminating echocardiographic features distinguishing Cpc-PH from Ipc-PH were RVFWS, RVFWS/PASP, TR velocity, FAC/PASP, septal e′ velocity, and LVEI.

In prognostic analyses using RF modeling followed by multivariable Cox regression, distinct echocardiographic predictors of mortality were identified between Ipc-PH and Cpc-PH phenotypes within our SSc-HFpEF population. Among SSc-HFpEF patients with Ipc-PH, mortality was associated with lower FAC/PASP ratio, increased LV mass, impaired LVGLS, higher pulmonary pressures, and reduced RVFWS. In contrast, mortality in the Cpc-PH group was linked to greater septal flattening as evidenced by higher LVEI, increased RVIDD, and more impaired biatrial reservoir strain. Reduced FAC/PASP was a shared predictor across both phenotypes. These findings underscore mechanistically distinct patterns of disease progression and highlight the pivotal role of RV-PA coupling, atrial dysfunction, and biventricular remodeling in determining outcomes in SSc-related postcapillary PH.

Despite increasing recognition of cardiac complications in SSc [1, 2], few studies have focused specifically on the postcapillary PH-LHD subtype in the context of HFpEF, and even fewer have examined echocardiographic markers capable of differentiating Cpc-PH from Ipc-PH. Data on phenotype-specific prognostic indicators in SSc-HFpEF remain particularly limited. Our study directly addresses these gaps by demonstrating that STE-derived parameters and RV-PA coupling metrics can discriminate between these hemodynamic phenotypes and provide prognostic information. Although the survival curves initially appeared similar, RMST analysis revealed significantly shorter long-term survival in patients with Cpc-PH, reflecting the adverse clinical implications of this phenotype. Collectively, these findings offer novel insights into the pathophysiologic spectrum of SSc-related PH-LHD and support a precision-based approach to phenotyping and management.

Prior work from our group demonstrated that, although survival appeared similar between patients with SSc-HFpEF-PH and those with SSc-PAH, the risk of death was approximately two-fold higher among those with PH-LHD after adjustment for hemodynamics [11]. The present study extends these observations by detailing the hemodynamic and structural severity of PH-LHD in SSc-HFpEF, characterized by elevated pulmonary pressures, right heart maladaptation, impaired RV-PA coupling, and derangements in atrial mechanics. These findings underscore that in SSc-HFpEF, right-sided maladaptation and pulmonary vascular dysfunction interact with left-sided diastolic disease, creating a compounded pathophysiologic burden that amplifies morbidity and mortality beyond conventional heart failure determinants.

We identified distinct echocardiographic features, including reductions in RV functional indices and hemodynamic abnormalities, that further differentiated postcapillary PH subtypes within our SSc-HFpEF cohort. Both RVFWS and RV-PA coupling measures (RVFWS/PASP, TAPSE/PASP, and FAC/PASP) were significantly lower in the Cpc-PH group, reflecting impaired right heart adaptation as a central pathophysiologic mechanism. Across WSPH classifications, the ability of the RV to adapt to increased PA afterload is a critical determinant of outcomes [22]. Echocardiographic indices of RV-PA coupling, such as the TAPSE/PASP ratio, have demonstrated prognostic value across diverse conditions [23–26], including pre- and post-capillary PH [27–29], and heart failure [30], and are endorsed by ERS/ESC guidelines [3] as validated noninvasive surrogates. In our SSc-HFpEF cohort, TAPSE/PASP was significantly lower in Cpc-PH (0.46 ± 0.25) compared with Ipc-PH (0.62 ± 0.32, *p* = 0.030), consistent with impaired RV-PA coupling and reduced contractile reserve under elevated afterload. These results align with prior work in non-SSc cohorts showing that lower TAPSE/PASP ratios reflect maladaptive RV remodeling and can identify higher-risk patients, including those with HFpEF [28]. 

However, TAPSE alone may be insufficient to distinguish adaptive from maladaptive remodeling [31], as it primarily reflects longitudinal annular excursion and susceptibility to both angle and translational effects [13]. Additionally, its prognostic accuracy may also vary with demographic and clinical factors such as age, sex, race/ethnicity, body habitus, comorbidities, and geometry of the right heart chambers [25, 32]. In contrast, indices incorporating FAC and RVFWS, may provide a more comprehensive assessment of RV mechanics by integrating both regional deformation and global cavity shortening under load [31–34]. Consistent with these results in our cohort RVFWS/PASP and FAC/PASP demonstrated stronger discriminatory ability and outcomes when compared to TAPSE/PASP, reflecting increased sensitivity to both regional and global contractility and coupling.

The present findings also align with prior work from our group. In SSc-PVD, we previously demonstrated that RVFWS/PASP and FAC/PASP outperform other coupling metrics for phenotypic classification [29], and improve risk score performance [14]. Similar relationships have been observed across broader non-SSc PVD populations, where RVFWS independently predicted mortality [33], and improved PAH risk stratification [35]. In non-PVD populations, we have also demonstrated that FAC/PASP accounts for sex- and age-specific geometric adaptation with physiologic aging in MESA [32], and correlates with clinical outcomes [25]. Moreover, we have shown that RVFWS is impaired in SSc even when conventional RV functional parameters are normal [36], and is further reduced in SSc-PAH compared to idiopathic PAH patients despite similar afterloads [37], supporting the presence of intrinsic myocardial dysfunction specific to SSc [38, 39]. The present study builds upon our prior work, demonstrating that reduced RVFWS and lower RVFWS/PASP ratios effectively distinguish Cpc-PH from Ipc-PH and predict adverse outcomes across subgroups. Additionally, reduced RVFWS was independently associated with mortality in Ipc-PH, while lower FAC/PASP predicted mortality across both phenotypes, underscoring that RV-PA uncoupling more accurately reflects global RV performance and prognosis than conventional systolic indices. Collectively, these data support the integration of coupling metrics into echocardiographic evaluation to refine classification, improve risk stratification, and inform mechanistically targeted therapeutic strategies in SSc-related PVD.

Among diastolic parameters, reduced septal e′ velocity independently associated with the Cpc-PH phenotype, consistent with advanced LV diastolic dysfunction and increased myocardial stiffness. The association supports the concept that chronic elevation in left-sided filling pressures in PH-LHD not only drives passive postcapillary transmission but may also contribute to active pulmonary vascular remodeling [40, 41]. Interventricular mechanical abnormalities further differentiated the phenotypes, with greater end-systolic septal flattening and higher LVEI in Cpc-PH reflecting RV pressure overload and interventricular dependence. LVEI emerged as both a key discriminator and an independent predictor of mortality, consistent with prior data indicating that values exceeding 1.2 identify precapillary physiology and adverse outcomes [42]. 

There is emerging evidence supporting the crucial role of RA mechanics in right heart performance in PH. The size of the RA independently predicts prognosis in PH, with an RA area greater than 26 cm^2^ linked to increased one-year mortality [3]. Elevated RA volume (RAV) and reduced STE-derived reservoir RA strain have been shown to predict clinical deterioration and correlate with functional status and hemodynamic abnormalities in pre-capillary PH [43, 44]. We similarly observed significant differences in atrial mechanics across Cpc-PH and Ipc-PH phenotypes in SSc-HFpEF. Reduced RA reservoir strain was independently associated with Cpc-PH and predicted mortality within this subgroup (HR 1.09 per % decrease, 95% CI: 1.01–1.18, *p* = 0.036), consistent with prior findings linking RA mechanical dysfunction to impaired RV-PA coupling and clinical deterioration in SSc-PVD [29]. Similarly, reduced LA reservoir strain was independently associated with Cpc-PH and with adverse outcomes, extending prior work demonstrating early LA myopathy in SSc preceding overt systolic or diastolic dysfunction [45]. These results underscore the value of atrial strain as a sensitive biomarker of disease severity and as a potential noninvasive tool for risk stratification in SSc-HFpEF with postcapillary PH.

Collectively, our findings delineate two distinct yet overlapping phenotypes within SSc-HFpEF-related PH-LHD. In Cpc-PH, the convergence of RV dysfunction, RV–PA uncoupling, impaired atrial mechanics, interventricular interaction, and elevated pulmonary pressures suggests a maladaptive remodeling process, likely compounded by intrinsic myocardial and vascular fibrosis characteristic of SSc. In contrast, the Ipc-PH subgroup appeared to reflect more traditional left heart remodeling with higher LV mass index and reduced LVGLS, but also with evidence of reduced RVFWS that was associated with mortality. These observations suggest a potential mechanistic continuum in which progressive right-sided maladaptation evolves from left heart disease, culminating in the mixed hemodynamic profile that defines Cpc-PH. Recognizing this transition has critical implications for patient monitoring, referral, and therapeutic strategies in SSc, where timely identification of Cpc-PH may enable earlier intervention and improved outcomes. Our findings add to the growing body of evidence supporting integrated echocardiographic assessment that combines indices of chamber-level function and coupling to provide a comprehensive evaluation of cardiopulmonary interaction, enhance risk prediction in PVD [33, 35], and delineate the distinct maladaptive remodeling patterns characteristic of SSc-associated PVD [14, 29]. 

## Limitations

Several limitations should be acknowledged. First, as a retrospective single center study from a tertiary referral hospital, our findings may be subject to selection bias and may not be generalizable to broader SSc populations. The higher prevalence of advanced disease may have influenced cohort severity. Second, unequal group sizes, particularly the larger Cpc-PH subgroup, may have affected comparative analyses. Third, although strain was assessed using standardized vendor independent software, image quality occasionally limited measurement availability. Fourth, the inclusion of patients with a TTE-RHC interval of up to 12 months introduces potential variability, as hemodynamic and loading conditions may change over time with disease progression, treatment, or volume shifts. Future studies should aim to minimize the TTE–RHC interval to improve alignment between echocardiographic and invasive assessments. In addition, the subset undergoing exercise hemodynamic testing was relatively small, limiting statistical power to detect exercise-specific differences. Despite the use of machine learning and multivariable models, residual confounding remains possible, including the unmeasured effects of comorbidities such as interstitial lung disease or medication use. Finally, cause-specific mortality data were unavailable, precluding determination of whether deaths were directly attributable to cardiopulmonary disease, and echocardiographic data were limited to a single time point, preventing assessment of longitudinal remodeling or disease progression.

## Conclusion

In patients with SSc-HFpEF, the presence of Cpc-PH was associated with pronounced structural and functional abnormalities, including RV systolic dysfunction, impaired RV-PA coupling, and biatrial remodeling. Strain-derived indices of RV function, RV–PA interaction, and atrial mechanics provided incremental value for differentiating Cpc-PH from Ipc-PH and for identifying patients at highest risk of adverse outcomes. These findings suggest that maladaptive right heart remodeling in SSc reflects not only increased afterload but also intrinsic myocardial and vascular pathology that parallels the fibrotic and microvascular processes characteristic of SSc pathophysiology. Integration of advanced echocardiographic metrics enables more precise, mechanistically grounded phenotyping and facilitates noninvasive monitoring of disease progression. Integrative echocardiographic approaches may improve classification of PH-LHD, inform timing of invasive evaluation, and guide individualized therapeutic strategies in patients with SSc.

## Data Availability

The datasets used and/or analyzed during the current study are available from the corresponding author on reasonable request.
